# Expression of amyloid-β in mouse cochlear hair cells causes an early-onset auditory defect in high-frequency sound perception

**DOI:** 10.18632/aging.100899

**Published:** 2016-03-07

**Authors:** Yasuhiro Omata, Suganya Tharasegaran, Young-Mi Lim, Yasutoyo Yamasaki, Yasuhito Ishigaki, Takanori Tatsuno, Mitsuo Maruyama, Leo Tsuda

**Affiliations:** ^1^ Center for Development of Advanced Medicine for Dementia (CAMD), National Center for Geriatrics and Gerontology (NCGG), Obu, Aichi, Japan; ^2^ Division of Molecular and Cellular Biology, Kanazawa Medical University, Kanazawa, Ishikawa, Japan; ^3^ Department of Molecular Aging, National Center for Geriatrics and Gerontology, Obu, Aichi, Japan; ^4^ Presend address: Department of Occupational and Environmental Health, Graduate School of Medicine, Nagoya University, Nagoya, Aichi, Japan

**Keywords:** aging, inner hair cells, deafness, Alzheimer's disease, amyloid-β

## Abstract

Increasing evidence indicates that defects in the sensory system are highly correlated with age-related neurodegenerative diseases, including Alzheimer's disease (AD). This raises the possibility that sensory cells possess some commonalities with neurons and may provide a tool for studying AD. The sensory system, especially the auditory system, has the advantage that depression in function over time can easily be measured with electrophysiological methods. To establish a new mouse AD model that takes advantage of this benefit, we produced transgenic mice expressing amyloid-β (Aβ), a causative element for AD, in their auditory hair cells. Electrophysiological assessment indicated that these mice had hearing impairment, specifically in high-frequency sound perception (>32 kHz), at 4 months after birth. Furthermore, loss of hair cells in the basal region of the cochlea, which is known to be associated with age-related hearing loss, appeared to be involved in this hearing defect. Interestingly, overexpression of human microtubule-associated protein *tau*, another factor in AD development, synergistically enhanced the Aβ-induced hearing defects. These results suggest that our new system reflects some, if not all, aspects of AD progression and, therefore, could complement the traditional AD mouse model to monitor Aβ-induced neuronal dysfunction quantitatively over time.

## INTRODUCTION

There are approximately 15,000 sensory hair cells in the mouse inner ear cochlea [1]. The hair cells are anatomically and functionally divided into two distinct types, namely outer and inner hair cells [2]. All hair cells are located on a thin basilar membrane along the cochlea, and the perception of different sound frequencies is dependent on the localization of hair cells on this membrane [3]. Notably, hair cells in the basal region of the cochlea are remarkably sensitive to external stress inducers, including reactive oxygen species, ototoxic drugs, and excessive noise [4]. In particular, aging is highly associated with impairment of hair cells in the basal region of the cochlea. Age-related hearing loss (ARHL) is usually due to loss and/or loss of function of hair cells in this region, and these cells are responsible for the perception of high-frequency sound; therefore, changes in perception in the high-frequency range are the earliest indicator of ARHL [5, 6].

Aging is also a challenge for neuronal cells in the central nervous system; age accelerates the symptoms of age-related neurodegenerative diseases, including Alzheimer's disease (AD), Parkinson's disease, and Huntington's disease [7, 8]. AD is an epidemic brain disorder characterized by memory deficits, spatial disorientation, and other psychiatric problems in older people [9]. Amyloid precursor protein (APP) is thought to be one of the key agents in AD development [9]. The truncated peptide amyloid-β (Aβ) is produced from APP by sequential proteolysis by β-secretases (cysteine proteases and β-site APP-cleaving enzyme) and γ-secretase (a multimeric protein complex composed of presenilin, nicastrin, Aph-1 and Pen-2) [9, 10]. Aβ appears to produce neuronal toxicity through binding neuronal receptors, such as the prion-receptor, metabotropic glutamate receptors or N-methyl-d-aspartate receptors [11-13].

Many studies have shown that defects in sensory systems are highly correlated with age-related neurodegenerative diseases, including AD [14-18]. This raises the possibility that sensory neurons have some commonalities with neurons in the brain. In support of this hypothesis, it has been shown that hair cells in the inner ear cochleae have properties similar to those of neurons: they electronically signal incoming auditory stimuli, make afferent and efferent synaptic connections to the auditory nerves, and the connectivity between hair cells and auditory nerves is important for sound perception [19]. Hair cells contain ion channels, including calcium-activated SK2 channels coupled to α9α10 nicotinic acetylcholine receptors, whereas the pre-synaptic region of auditory neurons contains many types of glutamate receptors, including N-methyl-d-aspartate receptors [19-21]. Furthermore, it was recently reported that the liver kinase B1, which is involved in cellular events in several organs including the central nervous system and mediates sporadic AD progression, is required for the maintenance of stereo cilia in the inner ear hair cells [22, 23]. These reports raised an intriguing idea that the auditory system may provide a useful tool to examine the development and progression of AD.

Auditory system function can be monitored using electrophysiological methods, which provides an easy way to observe functional impairment of sensory cells over time [19]. To develop a new AD model that takes advantage of this aspect of the auditory system, we established transgenic (Tg) mouse lines in which Aβ derivatives are expressed in the cochlear hair cells. Electrophysiological assessment of the auditory brainstem response (ABR) revealed that these mice had impaired high-frequency sound perception at 4 months after birth. We also observed that there was a synergistic interaction between *Aβ* and *tau* in the auditory system. In addition, we found that there was loss of hair cells in the basal region of the cochlea. Our data indicate that Aβ expression in the auditory hair cells induces early-onset auditory defects, demonstrating that this system provides an easy and quick assessment of Aβ-dependent toxicity, making it an ideal platform with which to monitor the effect(s) of Aβ-induced neuronal dysfunction over time.

## RESULTS

### Establishment of Tg mouse lines expressing *Aβ42* or its derivative in hair cells of the cochlea

**Figure 1:**
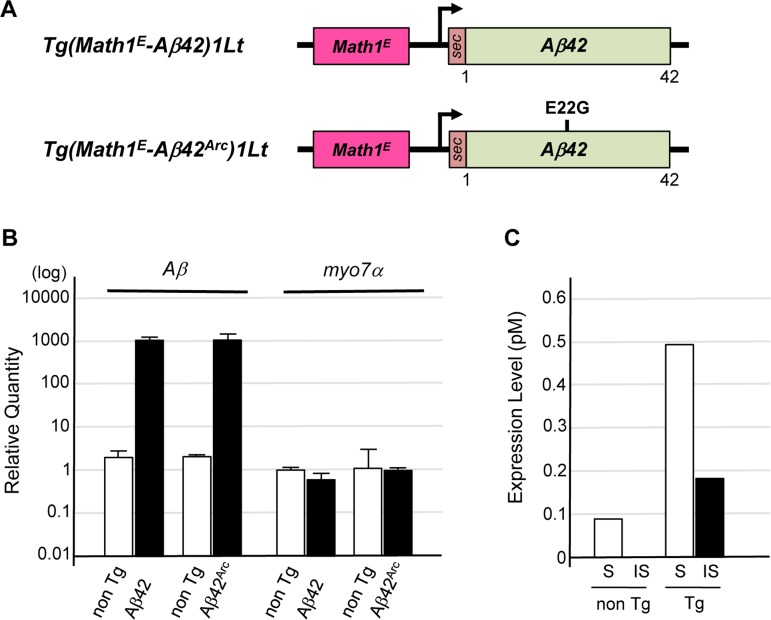
Establishment of Tg mice expressing *Aβ* in cochlear hair cells (**A**) Representative vector construction for the Tg mice used in this study. *Tg(Math1^E^-Aβ42)1Lt* contains *Aβ42* fused to the rat prepro-enkephalin secretion signal (sec) under the control of the *Math1* minimal enhancer (*Math1^E^*) and cytomegalovirus promoter. *Tg(Math1^E^-Aβ42^Arc^)1Lt* contains almost the same sequence as *Tg(Math1^E^-Aβ42)1Lt* except that the *sec-Aβ42^Arc^* sequence replaces *sec-Aβ42*. (**B**) Levels of *Tg(Math1^E^-Aβ42)1Lt* and *Tg(Math1^E^-Aβ42^Arc^)1Lt* mRNAs from cochleae were measured by qPCR and normalized to the control (*b-actin*) in non-Tg mice. (**C**) ELISA of cochleae extracts from *Tg(Math1^E^-Aβ42^Arc^)1Lt* and non-Tg mice at age 4 months. S; Tris-buffered saline–soluble form, IS; insoluble (formic acid–soluble) form.

To express *Aβ* or its truncated or mutated forms in mouse cochlear hair cells, we established Tg mouse lines carrying an expression vector for *Aβ*, a truncated form of *Aβ* (*Aβ42*), or *Aβ* with a familial AD mutation (*Aβ42^Arc^*, E22G substitution) downstream of the minimal enhancer region of mouse *Math1* (*Math1^E^*), which drives gene expression in many types of sensory neurons including cochlear hair cells [24] (Fig. 1A). These constructs were used for expression in mouse cochlear hair cells. The rat prepro-enkephalin signal sequence was fused upstream of the *Aβ* sequences to ensure their secretion [25]. Quantitative PCR (qPCR) showed that *Aβ42* and *Aβ42^Arc^* mRNAs were well expressed in the cochleae of Tg mice compared with non-Tg control mice (Fig. 1B). Enzyme-linked immunosorbent assay (ELISA) revealed the presence of Tris-buffered saline–soluble and –insoluble (formic acid–soluble) forms of an Aβ protein in the cochleae of 4-month-old *Tg(Math1^E^-Aβ42^Arc^)1Lt* mice (Fig. 1C).

### Aβ42^Arc^ is found around the hair cells of Tg mouse

**Figure 2:**
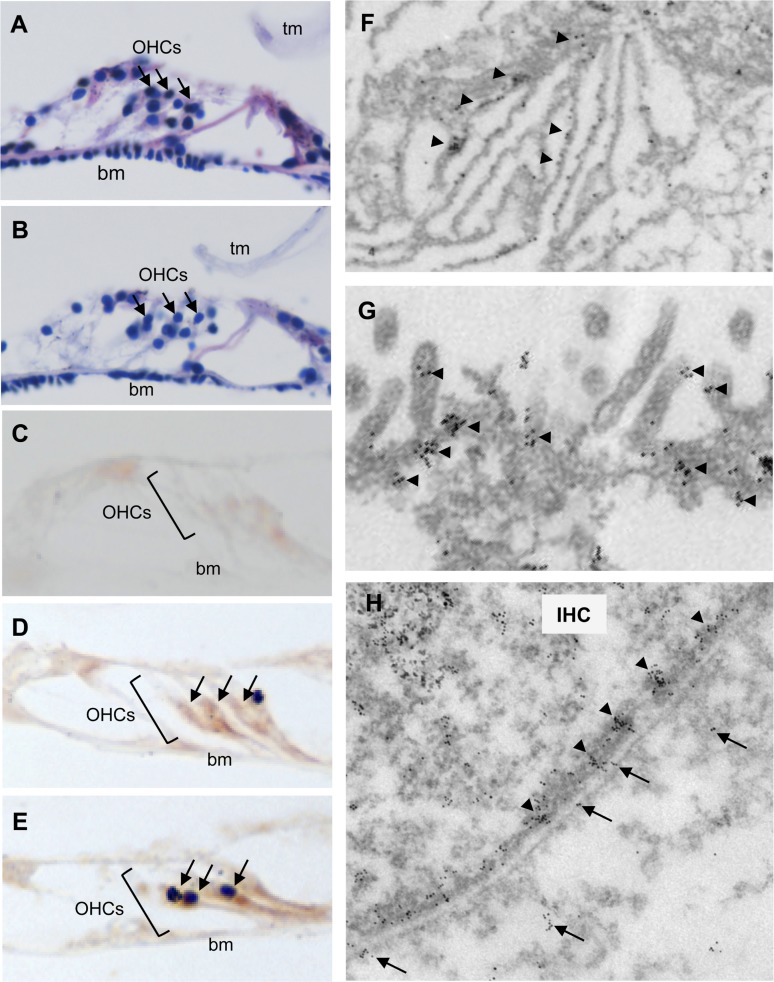
*Aβ42^Arc^* is expressed along the hair cells in the cochlea Hematoxylin and eosin staining of sectioned cochleae from 4-month-old non-Tg (**A**) and *Tg(Math1^E^-Aβ42^Arc^)1Lt* mice (**B**). OHC, outer hair cell (arrows); tm, tectorial membrane; bm, basilar membrane. (**C**-**E**) Immunostaining of cochlear sections at age 4 months using Aβ antibody (6E10). (**C**) Antibody 6E10 did not stain the cochlea of non-Tg mice. (**D**) The cochlear OHCs from *Tg(Math1^E^-Aβ42^Arc^)1Lt* were strongly stained by 6E10 (arrows). (**E**) Possible aggregation of an Aβ-like protein (arrows) in the cochleae of *Tg(Math1^E^-Aβ42^Arc^)1Lt* mice. (**F**-**H**) Immunoelectron microscopy of hair cells in *Tg(Math1^E^-Aβ42^Arc^)1Lt* cochlea at age 4 months. An Aβ-like protein accumulated in the endoplasmic reticulum (**F**), stereo cilia (**G**), and plasma membrane (**H**). Arrowheads: clusters of Aβ-like protein were observed around the stereo cilia (**G**) and plasma membrane (H). IHC: inner hair cell. Arrows: Aβ-like protein were also observed within the neighboring cells (**H**).

We surveyed the cochlear hair cells of *Tg(Math1^E^-Aβ42^Arc^)1Lt* mice histochemically and found that the structures of hair cells of Tg*-*mice were not different from those of non-Tg siblings at age 4 months (Fig. 2A, B). Next, staining with anti-Aβ (6E10) revealed that Aβ proteins were distributed mainly around the cochlear hair cells of 4-month-old *Tg(Math1^E^-Aβ42^Arc^)1Lt* mice (Fig. 2C, D). Aggregated Aβ proteins were also found near these hair cells, although they were not as abundant (Fig. 2E, arrows). To identify the precise cellular location of Aβ in the *Tg(Math1^E^-Aβ42^Arc^)1Lt* mice, we performed immunoelectron microscopy. Aβ immuno-staining was observed in the endoplasmic reticulum, the stereo cilia, and the plasma membrane of the hair cells (Fig. 2F, G and H). Notably, Aβ was found as clusters comprised of 2–20 particles in the stereo cilia and plasma membrane (Fig. 2G, H, arrowheads). This is similar to previous results that Aβ tends to exist as oligomeric clusters in the central nervous system [26]. Interestingly, Aβ immunostaining was also observed within and on the surface of the surrounding cells, suggesting that Aβ-like protein might be secreted to the cells neighboring the hair cells (Fig. 2H; arrows). These finding are in agreement with previously observed properties of Aβ-related peptides in the central nervous system [25, 27, 28].

### Expression of Aβ derivatives in cochlear hair cells impairs the perception of high-frequency sound

**Figure 3:**
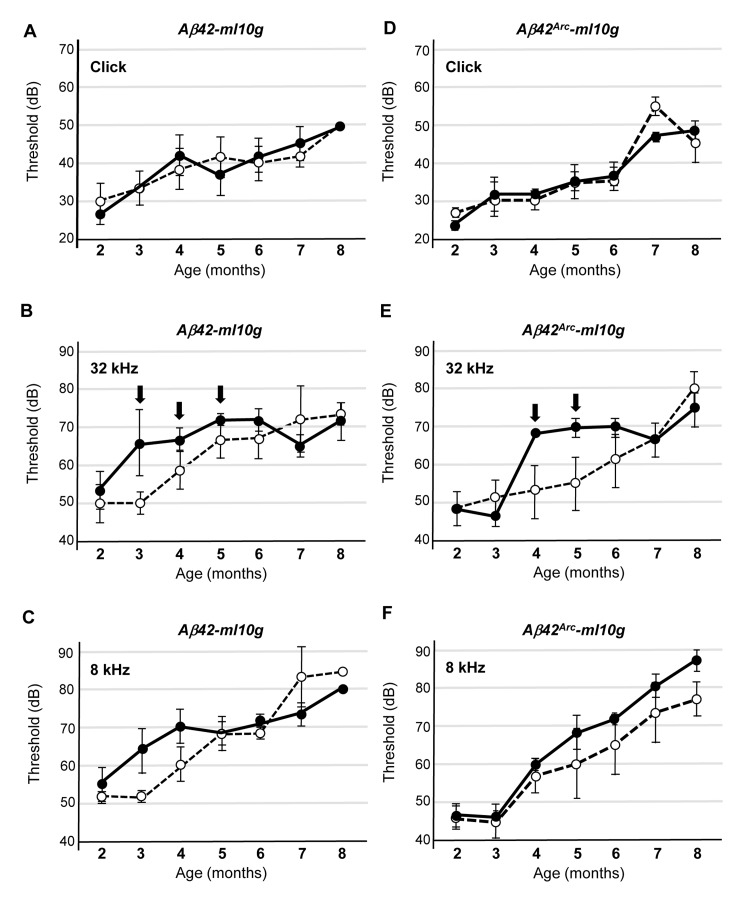
*Tg(Math1^E^-Aβ42)1Lt* and *Tg(Math1^E^-Aβ42^Arc^)1Lt* mice show slight hearing defects in high-frequency sound perception (**A**-**C**) ABR thresholds of *Tg(Math1^E^-Aβ42-ml10g)1Lt* (filled circles) and non-Tg (open circles) mice from age 2 to 8 months for the click sound (A), the 32 kHz tone (**B**), and the 8 kHz tone (**C**). ABR thresholds were slightly increased from age 3 to 5 months for the 32 kHz tone (B) (arrows). Non-Tg: n = 8 (5 male, 3 female), Tg: n = 10 (6 male, 4 female). (**D**-**F**) ABR thresholds of *Tg(Math1^E^-Aβ42Arc-ml10g)1Lt* (filled circles) and non-Tg (open circles) mice from age 2 to 8 months for the click sound (**D**), the 32 kHz tone (**E**), and the 8 kHz tone (**F**). ABR thresholds were slightly increased from age 4 to 5 months for the 32 kHz tone (**E**) (arrows). Non-Tg: n = 6 (5 male, 1 female), Tg: n = 10 (5 male, 5 female).

To assess the consequence of *Aβ42* or *Aβ42^Arc^* expression in cochlear hair cells, we monitored hearing with ABRs. ABRs are electrical action potentials in the cochlea and brain induced by an auditory stimulus and can be detected via electrodes placed on the scalp [29]. We monitored threshold for the first peak of the ABR response (1–2 ms after sound stimulation) because the first peak is generated exclusively by the activity of sensory hair cells and auditory nerves [19]. We first examined the response to a click sound, which is not frequency tunable but reflects the activity in a broad region of the cochlear hair cells, and we compared Tg mice and non-Tg mice that were wild-type littermates of the Tg mice. We found that the sound threshold for the non-Tg, *Tg(Math1^E^-Aβ42)1Lt*, and *Tg(Math1^E^-Aβ42^Arc^)1Lt* mice were not significantly different from age 2 to at least 8 months, suggesting that sound perception itself was not affected by the expression of *Aβ* in the hair cells (Fig. 3A, D). Next, we monitored the response to a high-frequency tone (32 kHz) and found that the thresholds for each of the *Tg(Math1^E^-Aβ42)1Lt* and *Tg(Math1^E^-Aβ42^Arc^)1Lt* mice was slightly increased from age 3 to 5 months (Fig. 3B, E, arrows), whereas the mid-range frequency (8 kHz) threshold of the *Tg(Math1^E^-Aβ42)1Lt* and *Tg(Math1^E^-Aβ42^Arc^)1Lt* mice was comparable to that of the non-Tg siblings during this period (Fig. 3C, F).

Although there were apparent differences in the ability of Tg mice to hear high-frequency sound, the ABR threshold increased with age for all mouse strains, and after the mice were 6 months old, we could no longer detect a difference between non-Tg and Tg mice.

**Figure 4:**
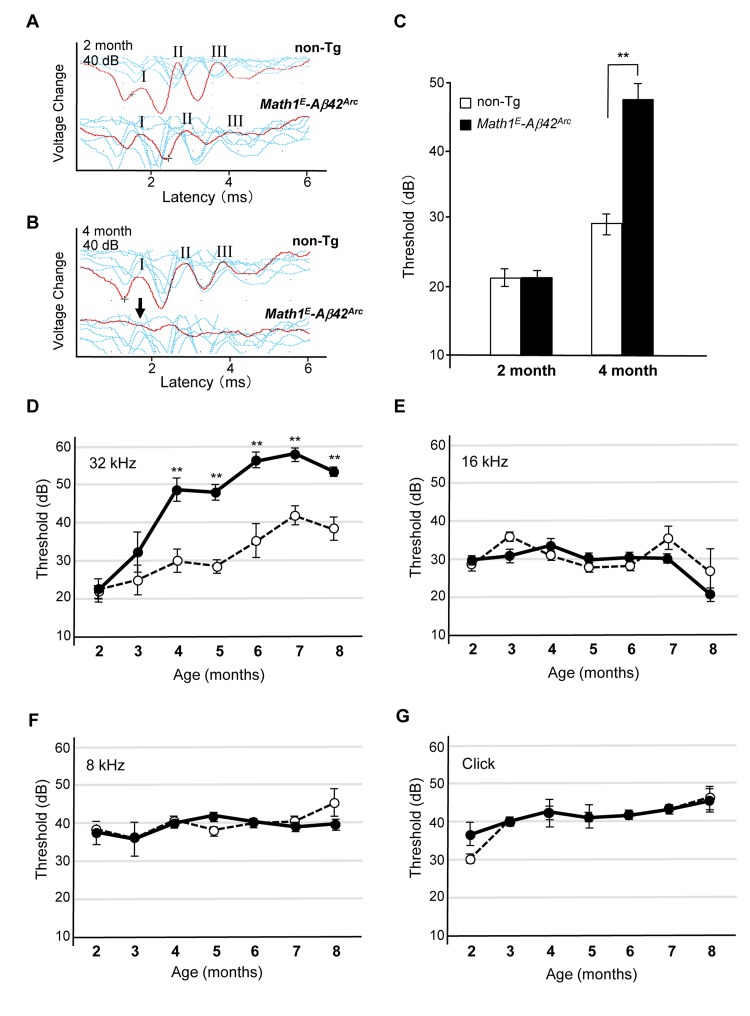
*Tg(Math1^E^-Aβ42^Arc^)1Lt/129* mice showed dramatic hearing impairment for high-frequency sound over time (**A**) Representative ABR data (Red line) of non-Tg (B6/129) and *Math1^E^-Aβ42^Arc^* (*Tg(Math1^E^-Aβ42^Arc^)1Lt/129*) mice in age 2 month for the 32 kHz at 40 dB. There were at least three peaks (I–III) in both genotypes (see METHODS). (**B**) Representative ABR data (Red line) of non-Tg (B6/129) and *Math1^E^-Aβ42^Arc^* (*Tg(Math1^E^-Aβ42^Arc^)1Lt/129*) mice in age 4 month for the 32 kHz at 40 dB. No first peak was observed in *Math1^E^-Aβ42^Arc^* mice (arrow). (**C**) ABR threshold of non-Tg (B6/129) (white bar) and *Math1^E^-Aβ42^Arc^* (*Tg(Math1^E^-Aβ42^Arc^)1Lt/129*) (black bar) mice in age 2 and 4 months for the 32 kHz at 40dB. (**D**-**G**) ABR threshold of *Tg(Math1^E^-Aβ42^Arc^)1Lt/129* and non-Tg (B6/129) mice from age 2 to 8 months for the 32 kHz tone (D), 16 kHz tone (**E**), and 8 kHz (**F**), and click sound (**G**). *Tg(Math1^E^-Aβ42^Arc^)1Lt/129* mice (filled circles) showed increasing ABR threshold compared with non-Tg (open circles) (**D**). No increase in ABR threshold was observed for the 16 kHz tone (**E**), 8 kHz tone (**F**), or click sound (G). Data represent the mean ± SEM. non-Tg: n = 4 (3 male, 1 female); Tg: n = 4 (2 male, 2 female). **P < 0.01.

The confluence of threshold might be a consequence of the genetic background of the Tg mice, which is C57BL/6 (B6). B6 mice have a point mutation in Cadherin 23, which induces an age-related hearing defect that leads to an increased dB threshold after age 6 months (Supplemental Fig. S1) [30, 31]. It has been shown that B6 and 129/SvJ (129) heterozygotes do not suffer from this age-related hearing defect [32]. Thus, we crossed *Tg(Math1^E^-Aβ42^Arc^)1Lt* mice with the 129 line and measured ABR of the heterozygotes (*Tg(Math1^E^-Aβ42^Arc^)1Lt/129*). We first compared the response to high-frequency (32 kHz) tone sound stimulation at 2 and 4 months of age and found that hearing ability was improved in the B6/129 background; the heterozygotes responded to a 40 dB sound stimulation, whereas the B6 mice no longer responded to this stimulation at age 4 months (Fig. 4A, B, compare to Fig. 3). We found that both non-Tg (*B6/129*) and *Tg(Math1^E^-Aβ42^Arc^)1Lt/129* mice showed a similar response at age 2 months, whereas the first peak could no longer be detected in *Tg(Math1^E^-Aβ42^Arc^)1Lt/129* mice at 4 months (Fig. 4A, B, arrow).

We then determined threshold levels at age 2 and 4 months and found that the *Tg(Math1^E^-Aβ42^Arc^)1Lt/129* mice showed a greatly increased threshold relative to the non-Tg (*B6/12 9*) mice at age 4 months (Fig. 4C).

Next, we monitored changes in threshold level from age 2 to 8 months in *Tg(Math1^E^-Aβ42^Arc^)1Lt/129* mice and confirmed that the mice showed an increasing threshold for high-frequency (32 kHz) sound stimulation from age 4 to at least 8 months (Fig. 4D). The hearing impairment of *Tg(Math1^E^-Aβ42^Arc^)1Lt/129* mice seemed to be exclusively in high-frequency sound perception; the threshold of the *Tg(Math1^E^-Aβ42^Arc^)1Lt/129* mice did not change for perception of lower-frequency sound (16 and 8 kHz) or click sound (Fig. 4E–G). These results suggeste d that *Aβ42^Arc^* expression in the hair cells exclusively affected the ability of the mice to respond to high-frequency sound (>32 kHz). To confirm that the defect in *Tg(Math1^E^-Aβ42^Arc^)1Lt/129* mice was exclusively in high-frequency sound perception, we analyzed the threshold response to a 40 kHz tone at age 8 months and found that the threshold was still significantly higher for Tg mice (Supplemental Fig. S2).

### Expression of *Aβ* derivatives in the hair cells causes loss of hair cells in the basal region of cochlea

**Figure 5:**
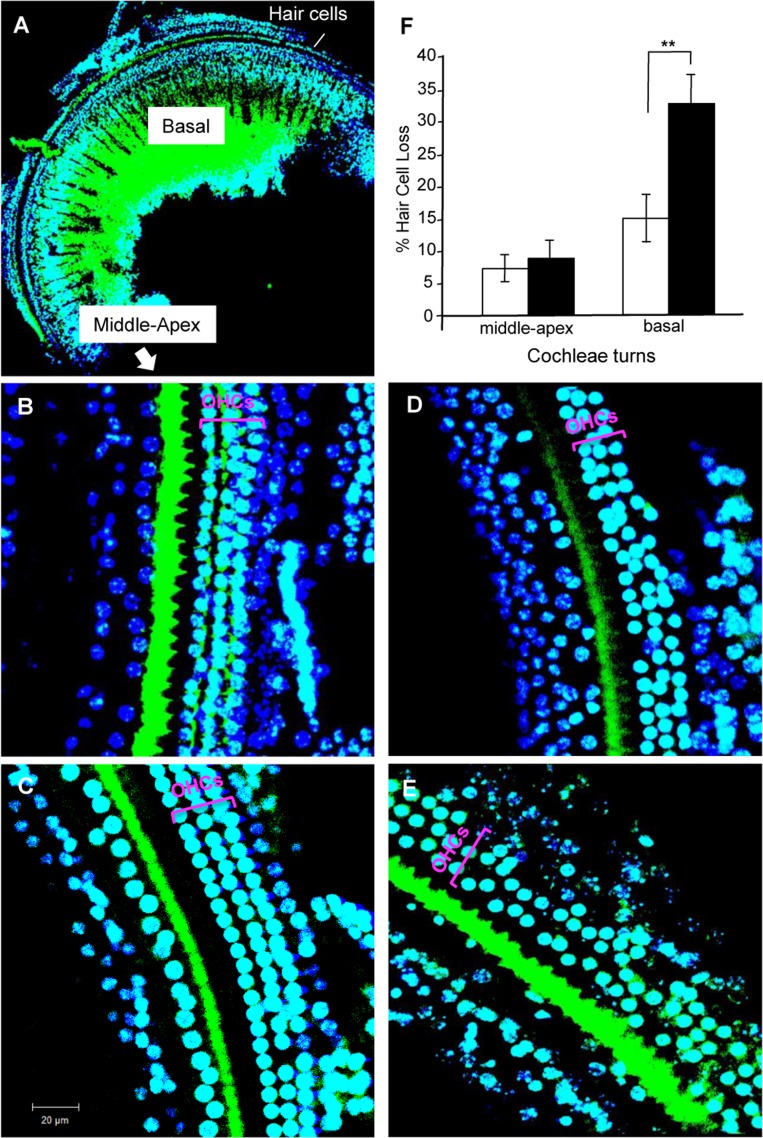
Hair cell loss in cochleae of *Tg(Math1^E^-Aβ42^Arc^)1Lt/129* mice and control non-Tg siblings at age 8 months (**A**) Cochleae of basal region staining with Alexa Fluor 488–phalloidin and DAPI. (**B**) Middle region of cochleae in non-Tg sibling. (**C**) Middle region of the cochlea in *Tg(Math1^E^-Aβ42^Arc^)1Lt/129*. (**D**) Basal region of the cochlea in non-Tg sibling. **(E**) Basal region of the cochlea in *Tg(Math1^E^-Aβ42^Arc^)1Lt/129*. OHCs, outer hair cells. The results of the OHCs count at each cochlea turns (% of hair cells loss) are shown in (**F**) (number of cochleae = 5, in each group). Middle-apex; middle to apex region. Basal; basal region. Data are the mean ± SEM. **P < 0.01.

High-frequency sound is perceived by the hair cells in the basal region of the cochlea [19]. Previous studies have suggested that the hair cells in the basal region of cochleae are lost during aging [4]. To elucidate the mechanism behind the defect in the ability to perceive high-frequency sound at age 8 months, we stained surface preparations of the organ of Corti derived from 8-month-old non-Tg and *Tg(Math1^E^-Aβ42^Arc^)1Lt/129* mice with Alexa Fluor 488–phalloidin and DAPI and visualized the hair cells (Fig. 5A). Non-Tg epithelia had one row of inner hair cells and three rows of outer hair cells at the basal, mid, and apical levels of the cochlear duct [1, 2]. We compared the hair cells in each region of the cochlea and found that the number of hair cells appeared to be comparable between non-Tg and Tg mice in the apical and middle regions of cochlea (the region responsible for perceiving frequencies <20 kHz) (Fig. 5A–C, F). However, the number of hair cells in the basal region of the cochlea (region responsible for perceiving frequencies >20 kHz) was dramatically reduced in the Tg mice (Fig. 5D–F). These results suggested that expression of *Aβ42^Arc^* specifically affects the survival of hair cells in the basal region of cochlea. This is correlated with the fact that neuronal cell death in the central nervous system is typical of AD pathology [33].

### Expression of the human microtubule-associated protein *tau* (*MAPT*) in cochlear hair cells exacerbates the hearing defects caused by *Aβ42^Arc^*

**Figure 6:**
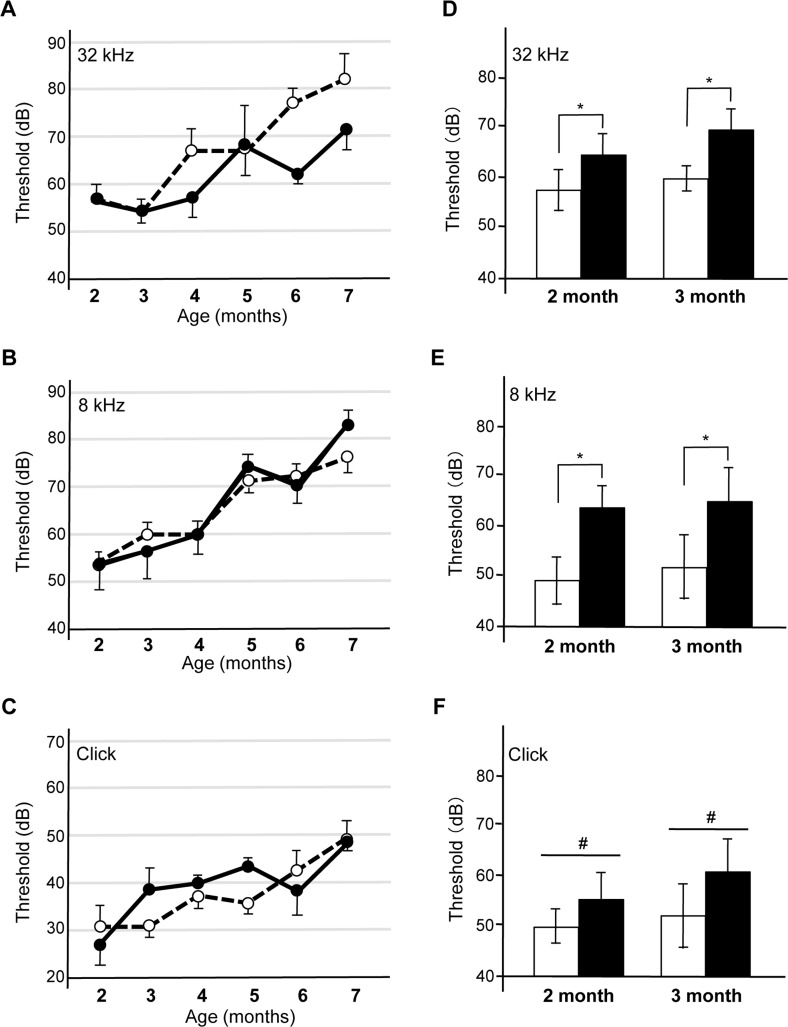
The expression of human tau in the cochleae of *Tg(Math1^E^-Aβ42^Arc^)1Lt mice* induces a synergistic auditory deficit (**A**-**C**) ABR threshold of *Tg(Math^E^-MAPT)1Lt* (filled circles) and non-Tg (open circles) mice from age 2 to 7 months for the 32 kHz (**A**) and 8 kHz **(B**) tones and the click sound (**C**). (**D**-**F**) ABR threshold for the non-Tg (white bars) and *tau/Aβ42^Arc^* (black bars) mice at age 2 and 3 months. *tau/Aβ42^Arc^* mice showed the threshold-increase onset at age 2 and 3 months for the 32 kHz (**D**) and 8 kHz (**E**) tones. The threshold for the click sound did not increase for any of the mice at any age (**F**). The data are expressed as the mean ± SEM. *p < 0.01; #, not significant. Non-Tg: n = 9 (6 male, 3 female); Tg (*tau*): n = 9 (4 male, 5 female); Tg (*tau/Aβ42^Arc^*): n = 5 (3 male, 2 female).

MAPT is thought to be another major player in the development of AD [34]. Many studies indicate that there are synergistic effects between *Aβ* and *tau* in the neuronal toxicity of AD in the brain [35, 36]. To elucidate if *Aβ42^Arc^*-dependent high-frequency hearing defect reflects other aspects of AD in the brain, we looked for genetic interaction between *Aβ42^Arc^* and *tau* in terms of hearing defects in the cochlear hair cells. We established another Tg mouse line, *Tg(Math^E^-MAPT)1Lt*, that expressed human *tau* (2N4R) in cochlear hair cells, and we monitored auditory function of these mice over time (Supplemental Fig. 3A, B). No significant defects in hearing were seen in these mice when exposed to sound frequencies of 32 or 8 kHz or to click sounds (Fig. 6A–C). To determine whether there is interaction between *Aβ42^Arc^* and *tau*, we established a double Tg mouse line (*Tau*/*Aβ42^Arc^*) harboring both *Tg(Math1^E^-Aβ42^Arc^)1Lt* and *Tg(Math^E^-MAPT)1Lt*. We found that these mice had severe hearing defects; the ABR threshold was significantly increased at age 2 and 3 months for both the 32 and 8 kHz tones (Fig. 6D, E). However, we did not detect an increase in the threshold for the click sound, suggesting that the hearing defect was only in the middle- to high-frequency sound perception (>8 kHz; Fig. 6F). The significant shift in the onset of the hearing defect from age 4 months in the *Aβ42^Arc^* mice to age 2 months in the *tau/Aβ42^Arc^* double Tg-mice strongly suggested that there is a synergistic interaction between *Aβ* and *tau* in this system.

## DISCUSSION

### The sensory system could be a useful tool to study age-related neurodegenerative disorders

It has been suggested that sensory impairments are involved in neurodegenerative disorders because olfactory defects appear to correlate with AD, mild cognitive impairment, Parkinson's disease, and Huntington's disease [15–18]. The auditory systems are also involved in the regulation of postural stability in AD patients [37], and there seems to be a correlation between deafness and AD-related dementia [38]. In mice, it has been shown that retinal expression of *Aβ* in *APPswe/PS1DeltaE9*, a mouse AD model, causes retinal dysfunction, suggesting that sensory decline with age-related neurodegenerative disorders might be evolutionally conserved [39]. In support of this notion, many studies have shown that compound eyes of *Drosophila melanogaster* can serve as a model for age-related neurodegenerative disorders such as AD, Parkinson's disease, and Huntington's disease [40–42].

Overproduction of the causative factors for those disorders in photoreceptor cells results in impaired survival of those cells. Notably, many of the molecules related to the pathological properties of these diseases in the brain were identified by genetic analysis using this system [43–45]. These results suggest that sensory system models for age-related neurodegenerative disorders might reflect certain aspects of neuronal defects in the brain.

In this study, we established a new AD model system that takes advantage of the mouse auditory system. We produced new Tg mice overexpressing *Aβ* or Aβ-related molecules in cochlear hair cells. The Tg mice showed auditory defects in high-frequency sound perception and synergistic interaction between *Aβ* and *tau* in terms of hearing impairment and hair cell loss (Figs. 4, 5, 6). Given that genetic interaction between *Aβ* and *tau* and loss of neuronal cells are typical pathological futures of AD [33, 34], we believe that our system reflects many, if not all, aspects of AD progression.

### Relationship between Aβ-induced hearing defects and ARHL

During aging, the survival of auditory hair cells decreases, especially in the basal region of the cochlea, and these cells are thought to be very sensitive to stressors such as excessive noise exposure [4]. This vulnerability of hair cells is likely the basis of the development of ARHL, as many animals with ARHL-related mutations show auditory defects after several hours of exposure to extreme noise [5]. We examined whether vulnerability of hair cells is involved in the hearing defects of *Tg(Math1^E^-Aβ42^Arc^)1Lt* mice by investigating if exposure to white noise at 100 dB for 2 h would affect the hearing ability of 3-month-old mice. One week after exposure, we measured the ABR threshold, however, no significant defects in hearing were seen in *Tg(Math1^E^-Aβ42^Arc^)1Lt* mice when exposed to sound frequencies of 32, 8 kHz or to click sounds (data not shown). Therefore, we think that auditory defects caused by Aβ differ from those associated with ARHL.

### *Tg(Math1^E^-Aβ42^Arc^)1Lt* mice are a potential new assay system for the study of AD

To investigate the toxic effects of Aβ during AD development, several different types of mouse models for AD have been established, many of which express human *APP* in the brain [46]. These *APP*-expressing mice are very useful as an assay system for senile plaque formation in the brain and for learning/memory impairment [46]. However, these *APP*-expressing mice have several disadvantages as a model system for the development of drugs against AD. First, it takes approximately one year to obtain the robust phenotype caused by Aβ toxicity. Second, the progression of disease in these systems is based on behavioral symptoms, which are assessed by a behavioral test such as the water maze or Barnes test [46]. Thus, these systems are very useful for measuring higher-order brain functions, such as learning and memory, but they are not well suited for quantitative analysis [46]. Third, *APP*-expressing mice do not show neuronal cell loss, which is a typical pathological feature found in AD patients [33, 34].

Herein, we describe a new Tg system to explore AD based on *Tg(Math1^E^-Aβ42^Arc^)1Lt* mice. Our Tg-mouse system can easily monitor the effect of Aβ toxicities using electrophysiological methods. In other words, we can assess the impact of Aβ in live animals quantitatively over time. In particular, this system can also detect cellular defects like hair cell loss when Aβ is expressed. Therefore, we expect that this system will be a powerful tool when it is complementary used with traditional AD mouse models for evaluating the effects of Aβ toxicity and developing therapeutic drugs against AD.

## METHODS

### Animals

Animal care and treatment were performed in accordance with institutional guidelines following approval by the Animal Care and Use Committee of National Center for Geriatrics and Gerontology (NCGG) (Obu, Japan). Before treatments, animals were anesthetized with isoflurane (Wako).

Mice were obtained from breeding colonies of *Tg(Math1^E^-Aβ42)1Lt*, *Tg(Math1^E^-Aβ42^Arc^)1Lt*, and *Tg(Math^E^-MAPT)1Lt*, which were heterozygous for the transgene, and were maintained at the Animal Center of NCGG. The Tg mice were maintained in a mixed C57BL/6 background and back-crossed to generate *Tg(Math1^E^-Aβ42)1Lt/B6*, *Tg(Math1^E^-Aβ42^Arc^)1Lt/B6* or *Tg(Math^E^-MAPT)1Lt/B6*. We refer to the resulting lines as *Tg(Math1^E^-Aβ42)1Lt*, *Tg(Math1^E^-Aβ42^Arc^)1Lt*, and *Tg(Math^E^-MAPT)1Lt*, respectively. Wild-type littermates of the Tg mice served as controls (non-Tg mice). The *Tg(Math1^E^-Aβ42^Arc^)1Lt* mice were also crossed with 129 to generate *Tg(Math1^E^-Aβ42^Arc^)1Lt/129*.

### Vector construction

We cloned the mouse *Math1* minimal enhancer (*Math1^E^*) sequence into a pBluescript plasmid (Stratagene) containing the cytomegalovirus promoter and IRES-EGFP::L10-β-globin heavy chain-poly(A) sequence [24]. *Aβ42* and *Aβ42^Arc^* constructs containing an upstream rat prepro-enkephalin signaling sequence, which had previously been constructed from cDNA of *APP* by PCR, or *MAPT* were placed downstream of the cytomegalovirus promoter [47, 48]. The mouse *Math1^E^* was kindly provided by Dr. J. E. Johnson (University of Texas Southwestern Medical Center, Dallas, TX).

### Real-time qPCR

The qPCR was performed as described [49]. Briefly, RNA was extracted from cochleae using Isogen (Wako) and the RNeasy Mini kit (Qiagen), and the different cDNAs were individually synthesized with Primescript RT reagent (Takara). The mRNA expression levels were quantified using a Thermal Cycler Dice Real-Time System and SYBR Premix Ex Taq (Takara). Data were normalized to *β-actin* mRNA expression level. Myo7a, unconventional myosins playing an important role in morphogenesis and organization of cochlear hair cell bundles, was used as a positive control. The thermal cycling parameters were 40 cycles at 95°C for 10 s and 60°C for 30 s.

Oligonucleotides for real-time PCR analysis were as follows.

*β-actin*:
  sense, 5′-CTAAGGCCAACCGTGAAAAG-3′
  antisense, 5′-ACCAGAGGCATACAGGGACA-3′
*Aβ*:
  sense, 5′-TCCGACATGACTCAGGATATGA-3′
  antisense, 5′-CCCACCATGAGTCCAATGA-3′
*myo7a*:
  sense, 5′-GCCATTGCTGACAACTGCTA-3′
  antisense, 5′-TTGTGCTCTCTGTCTTGCCA-3′

### ELISA

Soluble and insoluble (formic acid extractable) fractions were prepared from extracts of cochleae from *Tg(Math1^E^-Aβ42^Arc^)1Lt* and non-Tg mice. Briefly, frozen cochleae were homogenized with a metal pestle in Tris-buffered saline containing protease inhibitor cocktail (Complete Mini, Roche). After centrifugation at 100,000 × *g* for 1 h at 4°C, the soluble fraction was subjected to ELISA using the High Sensitivity Human β-Amyloid ELISA kit (Wako) to detect Aβ-like protein. The pellet was treated with 2% (w/v) sodium dodecyl sulfate and then 70% (v/v) formic acid and centrifuged at 100,000 × *g* for 1 h at 4°C. The supernatant, which represented the insoluble fraction, was subjected to ELISA.

### Histochemistry

Cochleae were isolated from *Tg(Math1^E^-Aβ42^Arc^)1Lt* and non-Tg mice at age 4 months and fixed in paraffin. Paraffin sections were stained with hematoxylin and eosin or immunostained using Aβ antibody 6E10 (BioLegend).

Epon-embedded tissue sections were prepared as described [47]. Briefly, dissected cochleae were fixed in 2.5% (v/v) glutaraldehyde and decalcified for 3 days at 4°C in 5 mM EDTA, 0.1 M sodium phosphate (pH 7.4). Tissues were then fixed with 1% (w/v) OsO_4_ (Wako) in PBS (pH 7.4) for 1 h at room temperature. Tissues were then washed with PBS for 2 h with the wash solution changed every 15 min and then dehydrated as follows: once for 15 min in 50% (v/v) ethanol, twice for 30 min in 70% (v/v) ethanol, and once for 30 min in 85% (v/v) ethanol. After treatment with propylene oxide (Nisshin EM Corporation.), tissues were embedded in Epon 812 resin (TAAB Laboratories Equipment.) and incubated at 60±C for 48 h.

Phalloidin staining was performed by the standard protocol [1]. Briefly, dissected cochleae were fixed overnight with 4% paraformaldehyde in PBS. After cochleae were decalcified for 3 days at 4°C in 5 mM EDTA, 0.1 M sodium phosphate, they were incubated with phalloidin antibody coupled to Alexa Fluor 488 (Thermo). Dissected cochleae were mounted with Vectashield Mounting Medium that contained DAPI (Vector laboratories).

### ABR measurement

ABRs were recorded in anesthetized mice. Electro-brainstem responses to the click (100 ms, 0–90 dB sound pressure level) and pure tonal sounds (8–32 kHz in half-octave steps, 20–100 dB sound pressure level in 5-dB steps, 3 ms, 1-ms cosine-squared rise-fall envelope) were recorded with subdermal silver wire electrodes at the ear (positive, active), the vertex (negative, reference), and the back of the mice (ground). ABR recordings were analyzed for consecutive amplitude deflections (waves). The first peak (wave I; latency, 1.2–1.9 ms) was monitored, and the hearing threshold for a given frequency or click stimulus was calculated for the lowest sound pressure leading to reliable ABR signals. Each ABR threshold was obtained by comparing the amplitude levels 5 dB above the hearing threshold for each group. Recordings were made for 12.5 ms with stimulus presentations of alternating polarity to eliminate electrical artifacts. In each case, stimulus presentation was at 0 ms. Signals were amplified (1000-fold, 40–60 dB), band pass filtered (0.2–5 kHz Butterworth, −20 dB/decade), averaged across 64–256 repetitions (dependent on the signal-to-noise ratio) at each sound pressure (usually 0–100 dB sound pressure level in steps of 5 dB), and recorded at 40 kHz sample frequency. Stimuli were delivered to the ear in a calibrated open system by a loudspeaker (model ES-1, TDT) placed 5 mm lateral to the pinna by an ear coupler. Sound pressure was calibrated online prior to each measurement with a microphone (NA42S, RION) placed under the scalp near the ear.

Mice were exposed to inhaled isoflurane anesthesia during ABR recording using a vaporizer.

### Data analysis

For statistical analyses, we used the Microsoft Excel Macro program (Microsoft) and JMP (SAS Institute).

## SUPPLEMENTAL FIGURES



## Abbreviations

ABR: auditory brainstem response
AD: Alzheimer's disease
Aβ: amyloid-β
APP: amyloid precursor protein
ARHL: age-related hearing loss
