# Supplementary material for: Expression of amyloid-β in mouse cochlear hair cells causes an early-onset auditory defect in high-frequency sound perception
Source: Aging (Albany NY). 2016 Mar 7;8(3):427–39. doi: 10.18632/aging.100899 (PMC4833138; doi:10.18632/aging.100899)
Supplement: Supplementary file 1 [file aging-08-427-s001.pdf]

SUPPLEMENTAL FIGURES

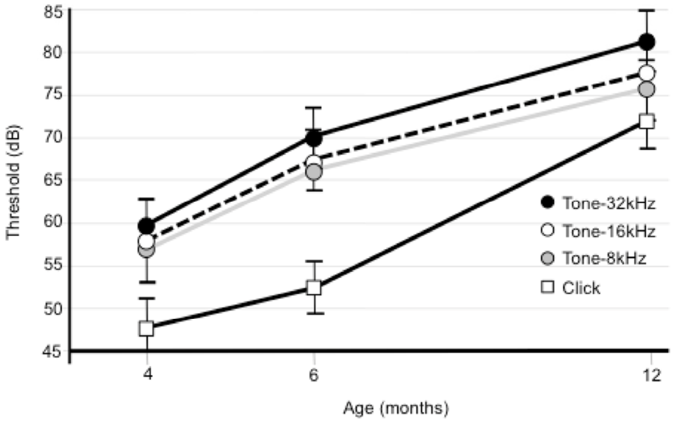

Supplemental Figure S1. Increasing ABR threshold with age in C57BL/6 mice. ABR threshold of C57BL/6 (B6) mice at age 2 to 12 months for the 32 kHz tone (black circles), 16 kHz tone (open circles), 8 kHz tone (gray circles), and the click sound (open squares). ABR threshold of C57BL/6 (B6) mice at age 2 to 12 months for the various tones and click sound. N = 4 (2 male, 2 female). Data represent the mean ± SEM.

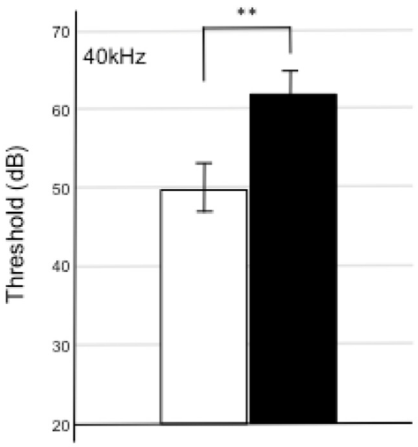

Supplemental Figure S2. Increasing threshold of 40 kHz sound perception at age 8 months. ABR threshold of 8-month-old *Tg(Math1^{E}-Aβ42^{Arc})1Lt/129* (black bar) and non-Tg (B6/129; white bar) mice for the 40 kHz tone. Data represent the mean ± SEM. Non-Tg: n = 4 (3 male, 1 female); Tg: n = 4 (2 male, 2 female). **P < 0.01.

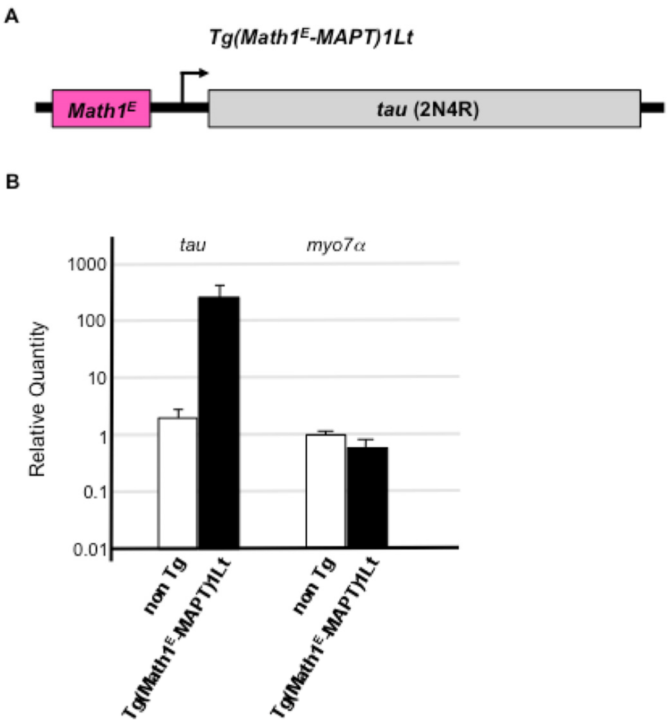

Supplemental Figure S3. Establishment of a Tg mouse line expressing MAPT in cochlear hair cells. (**A**) Representative vector construction for the Tg MAPT mice used in this study. *Tg(Math1^{E}-MAPT)1Lt* contains human *tau* (2N4R) under the control of the *Math1* minimal enhancer (*Math1^{E}*) and cytomegalovirus promoter. (**B**) Expression of *Tg(Math1^{E}-MAPT)1Lt* mRNA in cochleae was measured by qPCR and normalized to control (β -*actin*) in non-Tg mice.
